# Alignment of CanMEDS-Based Undergraduate and Postgraduate Pharmacy Curricula in The Netherlands

**DOI:** 10.3390/pharmacy8030117

**Published:** 2020-07-10

**Authors:** Andries S. Koster, Aukje K. Mantel-Teeuwisse, Herman J. Woerdenbag, Wilhelmina M. C. Mulder, Bob Wilffert, Tom Schalekamp, Henk Buurma, Ingeborg Wilting, Marnix P. D. Westein

**Affiliations:** 1 Department of Pharmaceutical Science, Utrecht University, David de Wiedgebouw, Universiteitsweg 99, 3584 CG Utrecht, The Netherlands; A.K.Mantel@uu.nl (A.K.M.-T.); T.Schalekamp@uu.nl (T.S.); M.P.D.Westein@uu.nl (M.P.D.W.); 2 Department of Pharmacy, University of Groningen, Antonius Deusinglaan 1, 9713 AV Groningen, The Netherlands; h.j.woerdenbag@rug.nl (H.J.W.); b.wilffert@rug.nl (B.W.); 3 Department of Clinical Pharmacy and Toxicology, Leiden University Medical Center, Albinusdreef 2, 2333 ZA Leiden, The Netherlands; W.M.C.Mulder@lumc.nl; 4 Royal Dutch Pharmacists Association (KNMP), Alexanderstraat 11, 2514 JL The Hague, The Netherlands; henkbuurma@ziggo.nl; 5 Department of Clinical Pharmacy, University Medical Center Utrecht, Heidelberglaan 100, 3584 CX Utrecht, The Netherlands; i.wilting@umcutrecht.nl

**Keywords:** competence-based pharmacy education, CanMEDS, curriculum design, expertise development, professional identity, integrated pedagogy model, self-determination theory, postgraduate specialization, community pharmacist, hospital pharmacist

## Abstract

In this article the design of three master programs (MSc in Pharmacy) and two postgraduate specialization programs for community or hospital pharmacist is described. After a preceding BSc in Pharmacy, these programs cover the full pharmacy education capacity for pharmacists in primary and secondary health care in the Netherlands. All programs use the CanMEDS framework, adapted to pharmacy education and specialization, which facilitates the horizontal integration of pharmacists’ professional development with other health care professions in the country. Moreover, it is illustrated that crossing the boundary from formal (university) education to experiential (workplace) education is eased by a gradual change in time spent in these two educational environments and by the use of comparable monitoring, feedback, and authentic assessment instruments. A reflection on the curricula, based on the principles of the *Integrative Pedagogy Model* and the *Self-determination Theory*, suggests that the alignment of these educational programs facilitates the development of professional expertise and professional identity of Dutch pharmacists.

## 1. Introduction

During the last few years of university training and the beginning of professional life pharmacy students have to make the transition from formal “academic” learning to practical “experiential” learning. At the same time, they have to develop professional expertise and a professional identity. This “boundary crossing” process can be complex [1,2]. Students must take increasing responsibility for their own learning, and the role of teachers becomes more and more facilitating, supporting, guiding, and coaching [3,4]. The nature of assessment (in a broad sense) is different: An important function of assessment is to provide students with feedback on their own learning (self-monitoring, assessment-for-learning) [5]. Frequently the same assessment results can be used for summative evaluation of student or trainee performance (assessment-of-learning) and for high-stakes decisions related to declaring a student or trainee “competent” [6]. At the end of this process the student/trainee is expected to be a reflective, self-directed pharmacy practitioner that is committed to life-long learning, is well-embedded in a professional “culture”, and that can bear independent responsibility for pharmaceutical patient care, in collaboration with patients and with other health care professionals [7,8]. 

Experiential learning has several characteristics that are different from academic learning. Tynjälä describes that *“Informal workplace learning is unplanned and implicit, often collaborative and highly contextualised, and the learning outcomes unpredictable, whereas school learning and organized on-the-job training is often formal, planned, largely explicit, focused on individual learning, and the outcomes are often predictable”* (ref. [9], p. 133). The tension between science-based and experience-based knowledge is also described by Waterfield [10] for pharmacy education. It is concluded that a close integration of science and practice is needed in pharmacy education [7]. Formal education is needed to obtain and develop generic skills that can be applied in various contexts, but in order to become a true expert a student has to develop situation-specific forms of competence, which only is possible in authentic learning environments.

**Figure 1:**
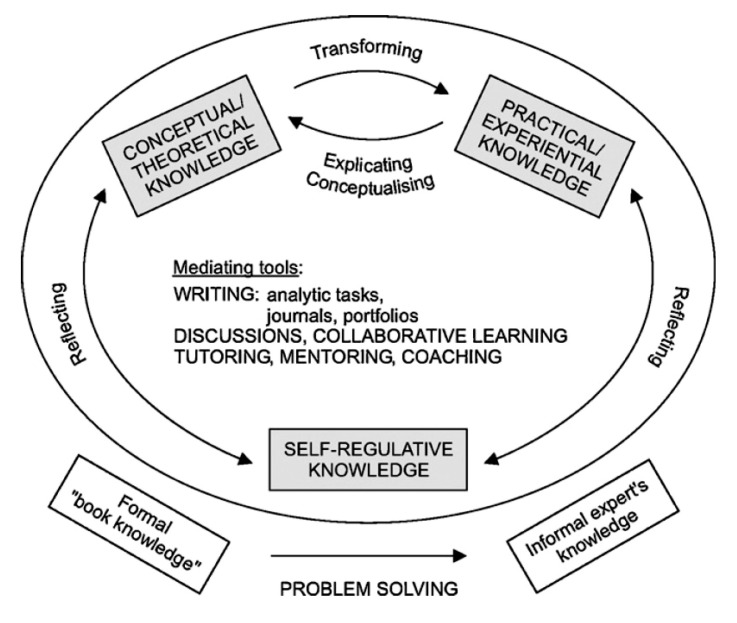
A model for development of professional expertise. Taken from ref. [9], with permission (nr. 4838831338331).

In the *Integrated Pedagogy Model* [9] three elements are considered essential for the development of professional expertise: theoretical/conceptual knowledge, practical/experiential knowledge, and self-regulatory knowledge. The development of professional expertise is seen as a process where theory cannot be separated from practice, and where practice cannot be separated from theory. Self-regulatory knowledge is needed to continuously reflect on the differences between both types of knowledge and to help in transforming theoretical knowledge into practical applications and to conceptualize practical experiences in terms of underlying concepts (Figure 1; see also ref. [11]). Several studies have described design principles for study programs that foster the development of self-directed and self-regulated learning at the boundary between higher education and work [12,13]. More explicitly, Elvira et al. [14]—based on the *Integrated Pedagogy Model* and an integrative literature review—have defined ten design principles, which support and foster the development of professional expertise [15]. These instructional principles are intended to facilitate transformation of theoretical/conceptual knowledge into practical/experiential knowledge (and vice versa) and to stimulate the use of self-regulation for reflecting on both practical and conceptual knowledge. Further details are given below (see Table 4). 

In addition to developing professional expertise the student or trainee is expected to be (and remain) motivated to become an expert and to develop professional identity. This is consistent with the societal expectations of the profession [7] and is reflected in competency-based frameworks such as CanMEDS. It has been suggested that the *Self-determination Theory* (SDT; ref. [16]) can be used to monitor the motivation of pharmacy students to develop their professional identity [17]. Recent studies have indeed shown that the SDT can be applied to monitor how motivation of practicing pharmacists [18] and pharmacist trainees (Westein et al., unpublished) is influenced by personal and workplace-dependent factors.

In this article we will examine the design of three different master programs and two postgraduate specializations (community pharmacist and hospital pharmacist) from the Dutch context through the lenses of the *Integrated Pedagogy Model* and the *Self-determination Theory*. We intend to illustrate how the design of these curricula, which are all based on an adapted CanMEDS framework, attempts to contribute optimally to the required development of professional expertise and identity.

## 2. Context: CanMEDS and The Dutch National Framework

Pharmaceutical health care in the Netherlands (17.4 million inhabitants) is provided in 1996 community pharmacies and 79 hospital pharmacies (including 8 academic hospitals). Community pharmacies are relatively large organizations and employ on average 1.6 pharmacists, 5.5 pharmacy technicians and 2.1 support personnel (year 2016, expressed as full-time equivalents, ref. [19]). Pharmacy technicians are involved in dispensing and patient counseling, under the pharmacists’ responsibility. Hospital pharmacies are even larger organizations and employ on average 10 pharmacists and 50 pharmacy technicians. A total of 2887 pharmacists work in a community pharmacy and 880 in a hospital pharmacy in 2019 [20,21].

**Figure 2:**
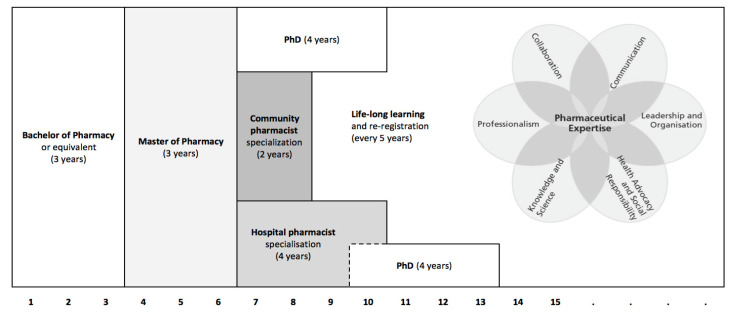
Dutch Pharmacy education. The curricula described in this article all use the CanMEDS model as a structuring principle. In this model professional competence is described in terms of the seven roles of Pharmaceutical expert, Communicator, Collaborator, Scholar, Health advocate, Leader and Professional.

Pharmacy education has a long tradition in the Netherlands. In addition to university degrees (BSc in Pharmacy and MSc in Pharmacy), two postgraduate specializations have been developed for the field of primary patient care (community pharmacy; since 1995) and secondary patient care (hospital pharmacy; since 1986). Gradual development of these programs has resulted in legal recognition of the “hospital pharmacist” specialization and the “community pharmacist” specialization in 1999 and 2016, respectively (Figure 2).

In 2005 the medical professions in the Netherlands adopted the CanMEDS model as a suitable framework to conceptualise educational programs for a diversity of medical specializations [22]. The CanMEDS model was also introduced as a basis for the updated versions of the hospital [23] and community [24] specialization programs (Table 1).

In 2016 two documents were published, both commissioned by the Dutch pharmacy departments in cooperation with the Royal Dutch Society of Pharmacy. The Domain-specific Frame of Reference for Pharmacy in the Netherlands describes the knowledge domain of pharmacy, the present and future developments in pharmacy, and defines the general tasks and responsibilities of the pharmacist. Moreover, it addresses the complementarity with physicians and the specific responsibilities of the pharmacist in pharmacotherapeutic treatment [25]. The Competency Framework (Table 1) specifies the required learning outcomes for pharmacists graduated from Dutch universities. The purpose of this framework was to guarantee the professional level of a starting pharmacist and to align the university’s bachelor and master degrees with the existing specialization programs in addition to being a future-proof framework that is in alignment with international developments [26,27] and the requirements of the Dutch law (Dutch Individual Healthcare Professions Act, BIG). In the European context it is, furthermore, required that during an undergraduate pharmacy program with a minimum duration of five years at least 26 weeks are spent in hospital and/or community pharmacies (EU directive 2005/36/CE; ref. [28]).

The CanMEDS model (Figure 2) distinguishes seven competency domains, which can be described as the roles of Pharmaceutical expert, Communicator, Collaborator, Scholar, Health advocate, Leader and Professional (see Table 1). Together, these roles cover the areas of responsibility in existing and future pharmacy practice (Product care, Patient care, Medication policy, Quality assurance and Research, education and innovation) as defined by the Dutch Individual Healthcare Professions Act (BIG). In the Competency Framework the competence level, required at the end of the master program, is defined for all competencies in terms of student independence and guidance by a supervisor (Table 1). For 92 out of 140 competencies the framework specifies that a graduated pharmacist “is expected to be able to adequately carry out professional activities in an authentic professional situation […] under supervision of an experienced pharmacy practitioner” (level V). This requirement particularly relates to the CanMEDS domains Pharmaceutical expert and Communication (see Table 1). In other CanMEDS domains (e.g., Interprofessional collaboration, Scientific research and Leadership) it is considered acceptable that recent graduates can function at a lower level of competency, requiring a more intensified level of guidance and/or supervision by experienced colleagues. Competence as such cannot be measured, but can be approached by assessment of behavioral proxies. Usually competencies have to be broken down in their constituent elements in the domains of knowledge, skills, and behaviour to be able to directly assess whether a student or trainee has mastered a particular competency (see ref. [29] for more details). Consequently, a variety of different assessment formats, suitable for assessing performance in authentic learning or training situations have been developed over the past decades (see Section 6 below and ref. [30]). More recently, the concept of Entrustable Professional Activities (EPAs; refs. [27,28]) has been introduced to structure training and assessments in competency-based medical education around those activities that are characteristic for the later professional life of a student or trainee. EPAs also are being considered as tools for structuring training and assessment of pharmacy students at the border between university and working life [29,30] and the use of EPAs has in fact been introduced in 2012 in the specialization program for community pharmacists in the Netherlands [31]. Currently the concept of EPAs is also being introduced as the backbone of the training program of hospital pharmacists.

## 3. Implementation of Pre- and Postgraduate Education

Translation of a set of required competencies into a curriculum is complicated [29,31]. Designing teaching/learning activities, feedback formats, and assessment tasks requires the adoption of an explicit model of student cognitive development [4,29,32] and alignment of all elements of the curriculum [33]. Mapping of the curriculum elements (teaching/learning tasks, internships, feedback formats, assessments, teacher, and preceptor roles) on a competency framework [34,35] can be helpful in identifying gaps, overlaps, omissions, and duplicates in the designed curriculum [36,37,38] and making comparisons between the experienced curriculum and the designed curriculum can be helpful in optimizing a curriculum [35,39]. Usually several rounds of internal and external reviews (visitation, accreditation) are required to arrive at an effective and efficient curriculum [29]. The curricula, described in this article, have gone through several rounds of internal and external review, either as part of a formal accreditation process by the Accreditation Organization of the Netherlands and Flanders NVAO (the master programs) or as part of internal reviews by professional organizations (Dutch Association of Hospital Pharmacists NVZA and Royal Dutch Pharmacists Association KNMP; see ref. [40]).

## 4. Three Different Master Programmes

Three universities in the Netherlands have a long tradition in carrying out pharmaceutical research and offering pharmacy training. In this article we will only discuss the 3-year Master in Pharmacy (MSc. Pharmacy) programs, but it should be noted that the same universities also offer undergraduate 3-year bachelor programs in pharmacy and pharmaceutical sciences (see endnote A). In September 2019 the annual intake of new students for the three master programs was 85, 140, and 50 in Groningen, Utrecht, and Leiden, respectively. In the Netherlands a license to work as a legally recognized pharmacist (Dutch Individual Health Professions Act) is automatically obtained with the master degree, followed by registration in the so-called BIG-register.

**Figure 3:**
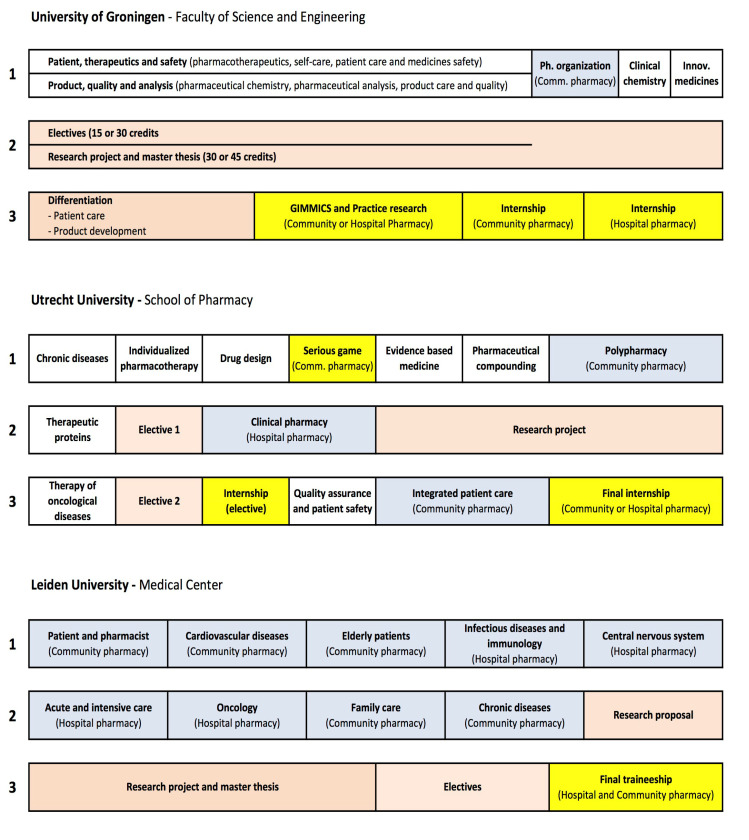
Three different master programs. All programs last three years (indicated as 1, 2, 3), during 40 weeks every year (left to right in the schemes). Colors indicate structured courses (white; obligatory for all students), individual subjects/electives (salmon), experiential internships (blue; 50–60% workplace) and full-time internships (yellow; at least 80% workplace).

Because all programs are based on a common competency framework (see above), they inevitably have common characteristics. However, at a first glance the curriculum structure appears very different (Figure 3). It can also be seen that the requirement of the European Union that at least 26 weeks are spent in a community and/or hospital pharmacy during the undergraduate training (EU-directive 2005/36/CE, ref. [28]), is implemented differently in the three programs.

The differences in curriculum structure partly represents historical developments. In Leiden a fundamental choice for introducing “experiential learning” was made recently (see endnote B) and this has resulted in a curriculum, where workplace learning is combined with formal learning to a large extent. In this system most teaching activities are organized around thematically organized experiential internships in a community or hospital pharmacy (e.g., Patient and pharmacist, Cardiovascular diseases). During a typical course, around 50–60% of the students’ time is spent in a pharmacy, the remainder being used for classroom meetings involving small-scale education and dedicated trainings. In Utrecht a similar course design is used for a large part of the required internships (Polypharmacy, Clinical pharmacy and Integrated patient care), while in Groningen this set-up is used for the introductory internship in year-1 (Pharmacy organization). Full-time internships, where more than 80% of the students’ time is spent at the workplace, usually are scheduled in the last year of the master programs (yellow colour in Figure 3). In Groningen and Utrecht serious gaming is used as an initial confrontation and training with the day-to-day complexities of pharmaceutical practice. In the pharmacy game Gimmics^®^ (Groningen Institute Model for Management in Care Services; Groningen, The Netherlands) groups of 5–8 students in their final year, have to organize and run a simulated community pharmacy [41]. In Utrecht a similar set up is used in the first master year. 

Article 44 of the EU-directive stipulates that the total duration of undergraduate education must be at least five years. Within Europe the Netherlands is exceptional in having a 6-year academic pharmacy program (i.e., a three-year bachelor and three-year master), as required by Dutch law. The scientific nature of the programs is emphasised by requiring that each student carries out an independent research project during one semester (see Figure 3). Together with a wide choice of electives this gives students the opportunity to follow a path of preference towards a more patient-oriented or product-oriented study program by choosing courses and research topics in different research departments within the same or other universities in the Netherlands or abroad. Only the University of Groningen recognizes a formal (but limited) “differentiation” between a Patient care and a Product development trajectory. Elective internships in pharmaceutical industries or regulatory authorities are allowed in all master programs, but no formal specializations are recognized within the master diploma. In general, students have a wide range of options to shape their own profile (at least 1/3 of their total master program). They have to be proactive in planning their own study program and in making arrangements with their research departments and study coordinators. Therefore, the trajectory of study of an individual student can differ considerably from the scheme in Figure 3, beyond the compulsory elements of the programs.

Part of the programs in Groningen and Utrecht consists of courses, which are obligatory for all students (white colour in Figure 3). In Utrecht these courses all last five weeks (full time) and are organized around relevant themes (e.g., Chronic diseases, Individualized pharmacotherapy and Drug design; see Figure 3). In Groningen disciplinary courses are organized under two large themes, Patient, therapeutics and safety (which includes general pharmacotherapeutics, self-care, patient care and medicines safety), and Product, quality and analysis (which includes pharmaceutical chemistry, pharmaceutical analysis, product care and quality). The students follow these courses in the first year of the master program.

## 5. Two Postgraduate Specializations

The two postgraduate specializations, for community pharmacist and hospital pharmacist, have similar characteristics (Table 2). Both programs are workplace based. Trainees, which are registered as pharmacists, are employed by a pharmacy or hospital and are further trained under the guidance of experienced colleagues. The main difference between the two programs is duration (two years versus four years), the number of training locations (one versus two) and the possibility for differentiation within the program. In the community pharmacist specialization program differentiation is limited to individual research projects, while in the hospital pharmacist specialization program trainees are expected to follow an individual differentiation, which is characterized by a research project in combination with science training courses (see endnote C). Some trainees in the hospital training continue into a formal PhD trajectory following their research project and after obtaining their registration as a hospital pharmacist (Figure 2). Alternatively, training for hospital or community pharmacist can start after finishing a PhD project.

In addition to workplace-based training, centralized courses are organized for all trainees. For trainees in the community pharmacist specialization program, courses on ethics, leadership, management skills, and relevant aspects of product care and pharmacotherapy are organized. Centralized courses for trainees in the hospital pharmacist specialization program in year-1 are generic and expand on pharmaceutical product knowledge, pharmacotherapeutic topics, toxicology and management aspects of the master programs. In year-2 to -4 specific courses in the area of differentiation (e.g., neurology, oncology, psychiatry, pharmacovigilance, research methodology) can be followed. In addition to contributing to specialised knowledge and skills, these centralized courses also contribute to the development of a community-of-practice because the trainees will follow these courses together with colleagues from other training locations.

Not all pharmacists in the Netherlands choose to specialize as either community or hospital pharmacist. After obtaining their master degree, and registration in the BIG-register, they are entitled to work in any position which requires a pharmacists’ license. Pharmacists working in education, regulatory or industrial positions often become specialized as well by specific research and or dedicated training programs.

## 6. Feedback, Assessment, and Monitoring

Competency-based education requires that skills are assessed on a regular basis, in addition to knowledge, to identify existing gaps in knowledge and to establish professional growth. Moreover, it is required that students will frequently receive feedback on their performance in authentic assessment situations [29,30]. Preferably a wide range of assessment types is used, which are not limited to the lower levels of Miller’s pyramid (“knows” and “knows how”; ref. [42]). Giving feedback on the higher levels “shows how” and “does” in authentic learning situations becomes increasingly important as students progress in the program(s). Recent literature reviews [30,43] have analysed the validity and reliability of various assessment formats, and these authors conclude that various workplace-based assessment formats have great potential in competency-based education (see Table 3).

Short practice observations (SPO), Directly observed preparation skills (DOPS), Case-based discussions (CBD), Critical appraisals of topics (CAT), and Multisource feedback (MSF), when used in real working situations, are highly authentic and have reasonable validity and reliability [30]. Most of these assessment formats can be adapted to (simulated) learning or training situations at the expense of full authenticity, which makes them suitable for use in earlier stages of student development, e.g., during the master program. The use of Objective structured clinical examinations (OSCE) is relatively well investigated [44,45].

In the master programs in Groningen, Utrecht and Leiden the CBD- and SPO- formats are used frequently for assessment, in addition to conventional multiple-choice and written (closed book or open book) exams; the OSCE-format is used to a limited extent. In almost all courses or internships a combination of various testing formats aims for a balanced assessment of knowledge, skills, and actual performance of students. Assessment in a typical course may consist of a diagnostic test at the start, a mid-course exam, an end-course written exam (with essay-questions) and assessment of patient-consulting or compounding skills with CBD-, SPO-, or DOPS-formats. All universities require that the relationship between intended learning outcomes and assessment formats in individual courses be described in an integrated assessment plan for the program. The integrated assessment plans of the three master-programs were evaluated as “sufficient” or “good” in a recent external evaluation by the Accreditation Organization of the Netherlands and Flanders (2019).

In the two postgraduate specializations, authentic assessment formats are the main feedback and assessment tools used. In addition, MSF is used as an instrument in both programs (see Table 2 for details). In the community pharmacist specialization program the format chosen is dependent on the Entrustable professional activity (EPA) being assessed [40]; in 2021 EPAs and Entrustment-based discussions (EBD, ref. [48]) will also be introduced in the hospital pharmacist specialization program as organizing principles.

In the three master programs and the specializations programs electronic portfolios (EPASS^®^, Mateum BV, Born, The Netherlands; Scorion^®^, Parantion Groep BV, Deventer, The Netherlands; or otherwise) are used as monitoring instruments. The results of required assessments and evaluations (Table 3) are recorded in an organized and traceable way for use in performance evaluations. In addition, the portfolio is used to collect individual work, personal development plans and reflection documents of students/trainees [57]. The portfolio is primarily maintained by the student/trainee, but supervisors and/or assessors are allowed to add feedback and performance evaluations. The portfolio functions as a central repository to record the competence development of the student or trainee. Integrated feedback on the competence development of a student or trainee is given in the form of Formative performance evaluations (FPE), where the functioning of a student/trainee in a work environment is evaluated on a regular basis (see Table 3). The results of other assessment formats, usually collected in the students/trainees’ portfolio, function as input for the FPE and the result is described in terms of the seven CanMEDS roles Pharmaceutical expert, Communicator, Collaborator, Scholar, Health advocate, Leader, and Professional (Figure 1 and Table 1). In the specialization programs for community and hospital pharmacist FPEs are used on a regular basis as a feedback and monitoring tool (every three months). In the master program of Utrecht University the FPE is used as an assessment tool during the internships in hospital and community pharmacies, while in Leiden FPEs are used in all experiential internships.

Performance evaluations can be used also as a basis for high-stakes decisions [6,56]. In the specialization programs these so-called Summative performance evaluations (SPE) are used to decide after one year whether a trainee is suitable for continuing with the program (intermediate SPE) and to decide at the end of the program whether the trainee can be registered as a community or hospital pharmacist specialist (final SPE; see Table 2).

## 7. Common Design Principles

Constructing an effective competency-based educational program requires a careful design, where all elements are aligned with each other both horizontally and longitudinally [58,59,60]. Horizontal integration means that disciplinary knowledge and disciplinary-specific skills become integrated in curriculum elements (courses, etc.). It is advised that integration increases gradually and that student assignments and tasks increase in complexity as the curriculum progresses [29,59]. In the presently described curricula different ways of integrating disciplinary content can be recognized (Figure 3). The first year of the master program of the University of Groningen consists of disciplinary courses, but these courses are scheduled under two overarching themes (Patient, therapeutics and safety and Product, quality and analysis, respectively) to facilitate integration of disciplinary knowledge and skills. Another way of integrating knowledge is by organizing courses around themes (rather than disciplines), which is illustrated by courses on Innovative medicines (Groningen, year 1) and many courses in the program of Utrecht University (e.g., Chronic diseases and Quality assurance and patient safety) and Leiden University.

Two other important aspect of a curriculum design are the progressive integration of content and skills across disciplines [29,59] and the transition from formal learning to experiential learning [9] as the curriculum progresses. In the described master programs training of skills starts in individual courses without having a direct connection with a working environment (e.g., a patient interview training in the course Chronic diseases of Utrecht University). In the experiential internships (indicated by the blue color in Figure 3) knowledge and skills are brought close to each other because students spend half of their time in a pharmacy environment, the remainder being spent in the university. A direct confrontation between conceptual ways of learning (university) and experiential ways of learning (pharmacy) is intended to stimulate the development of self-regulated learning [9,12]. A next step to experiential learning is made, when a student is spending most of the time in a pharmacy during the internships at the end of the master program (indicated by the yellow colour in Figure 3). In the specialization programs trainees are no longer considered “students”; they are employed as legally recognized pharmacists and will have responsibilities in important professional domains, be it under supervision of senior colleagues (Table 1). The work-based environment of the specialization programs for hospital or community pharmacist contributes importantly to the building of practical experience and to the elaboration of specialised knowledge and skills.

During the curricula described in this article the students/trainees typically progress from “advanced beginner” to “proficient” expertise; they are considered “competent” at the boundary between university education and specialization [15]. As the curriculum progresses the role of teachers is expected to change considerably [3]. At the beginning of the master program teachers will have considerably more knowledge and better skills than their students; at the end of a specialization program trainees will have become colleagues of their former teachers. For the teachers this means a shift from “knowledge provider” to “coach” and “critical friend”. In a recent systematic review commitment to teaching, role modelling, and encouragement of self-directed learning were identified as the three main competences for teachers/preceptors in advanced pharmacy practice education, in addition to having the knowledge and skills, relevant for their area of clinical work [61]. It is clear, therefore, that also teachers and supervisors, need to be able to “cross borders” between their pharmacy profession and their student/trainee guidance role on a regular basis. Teachers and supervisor in the specialization programs, described in this paper, are prepared for their role by regular training activities (Table 2). Selection and training of supervisors for the community pharmacist specialization program is guided by an explicit *Comprehensive pharmacist supervisor competence profile* [24], which forms the basis for a two-day training program.

Development of professional expertise not only takes time, but also requires specific training and coaching trajectories [9,15]. Several teaching and learning principles for effective development of expertise can be derived from the educational research literature [9,14]. The *Integrated Pedagogy Model* [9] posits that self-directed and self-regulated learning at the boundary between higher education and work is best developed when theoretical/conceptual knowledge and practical/experiential knowledge are confronted with each other and when self-regulation is used to reflect effectively on the differences between both types of knowledge. Based on this model, ten educational design principles, which are considered effective for transforming conceptual knowledge into experiential knowledge (design principles 1–5; see Table 4), for explicating experiential knowledge into conceptual knowledge (design principles 6 and 7) and for reflecting on practical and conceptual knowledge by using self-regulative knowledge (design principles 8–10) have been formulated [14]. In Table 4 it is illustrated how these principles are implemented in the master and specialization programs, described in this article. It can be seen that the major curriculum characteristics (courses, internships, assessment and monitoring tools) potentially facilitate the development of professional expertise. Nevertheless, a deeper level of analysis at the level of teaching/learning activities and teacher roles is required to evaluate whether the full potential of the curricula in this respect is reached. This is, however, beyond the scope of this article.

Formation of professional identity is strongly dependent on the experience of students/trainees during their internship placements [1,63]. Being a member of both learning communities (university and workplace) during prolonged time is considered to be essential for crossing the boundary between formal education and workplace-based learning and for the development of a context-specific professional identity [9,14,63]. Being able to observe how positive role models function in the working environment [2] and being exposed to purposeful and relevant learning activities [64] also contribute to the formation of a professional identity. In terms of the *Self-determination Theory* [16,17] the motivation of trainees to develop a professional identity depends on fulfilment of their basic psychological needs to experience competence, autonomy and relatedness (see Table 4). In the curricula, described in this paper, the gradual development of competence is structurally addressed by giving feedback based on the CanMEDS competency framework, which attends to competence development in all domains of professional life (see Table 1). By offering individual practice-oriented and/or research projects and differentiation options within the educational programs the need for autonomy is also addressed. The development of relatedness is facilitated by structuring teaching–learning activities as group efforts (collaborative or cooperative formats) with fellow students/trainees and by frequent interactions between students/trainees and their supervisors in feedback activities (see Table 4 for details).

## 8. Conclusions

In the Netherlands the undergraduate programs (MSc. Pharmacy) and the postgraduate specializations for hospital or community pharmacist use the CanMEDS model as a backbone. The CanMEDS competencies have been adapted from medical expertise to the development of pharmaceutical expertise. Using the same framework facilitates the alignment of the different programs with each other and enables interaction with other health care professional education programs, such as the programs for medical specialists [22], including general practitioners [65]. This article also illustrates that the structure of three master programs at a first glance may seem different, even though they are based on the same framework. Furthermore, it is shown that crossing the boundary between formal (university) learning and experiential (workplace) learning is eased by applying common design principles, both in the use of teaching–learning activities and in the use of assessment procedures. A gradual integration of disciplinary content, integration of skills with content, increasing complexity of assignments/problems, and a gradual change in the time spent in the formal and experiential learning environments are supposed to “blur” the boundary between university education and professional life. By making a comparison with established theoretical models for the development of professional expertise and professional identity it is shown that all programs contain design elements that are supposed to contribute to these two important aspects of the professional life of a community or hospital pharmacist. 

## Endnotes

- BSc. Pharmacy programs are available in Groningen and Utrecht and separate programs in pharmaceutical sciences are offered in Utrecht (College of Pharmaceutical Sciences) and Leiden (BSc. Biopharmaceutical Sciences). Master programs in pharmaceutical sciences are available in Groningen (MSc. Medical Pharmaceutical Sciences), Utrecht (MSc. Drug Innovation) and Leiden (MSc. Biopharmaceutical Sciences).
- This program started in 2016 after a more traditional pharmacy program was discontinued in 1985 following a decision of the Dutch government.
- Trainees have to choose between a formal training program in Epidemiology, Clinical pharmacology or Clinical toxicology, a differentiation in the area of compounding, radiopharmacy or laboratory sciences, or a pharmacotherapeutic differentiation in clinical areas such as geriatrics, infectious diseases, intensive care, oncology, pediatrics or psychiatry. The differentiation is described in a personal development plan, which needs to be accepted by the Specialist Registration Commission.

## Figures and Tables

**Table 1:** Required competency levels at the end of the master program.

CanMEDS Domains (Role)	Number of Competencies	Level Required
1. Pharmaceutical expertise (Pharmaceutical Expert)		
generic knowledge and skills	3	V
responsibility for product care	11	V
responsibility for patient care	15	V
responsibility for medication policy	3	III, IV or V
handling of scientific information	5	V
interprofessional communication	2	V
self-reflection, morality and ethics	5	V
2. Communication (Communicator)		
pharmacotherapeutic relationship with patients, carers	5	V
collecting relevant patient information	2	V
interprofessional communication	4	V
pharmaceutical supervision of patients	3	V
special patient groups: children and elderly, multiculturality	3	V
providing verbal and written feedback	5	V
3. Interprofessional collaboration (Collaborator)		
development of pharmacotherapy treatment plan	2	IV
contribute effectively to interprofessional teams	6	III or V
contribute to quality of organization	2	III
4. Scientific knowledge and research (Scholar)		
conduct relevant scientific research (research cycle)	7	III
educational activities	4	IV
critical evaluation of literature	5	V
evidence-based decision making	4	V
personal learning strategy	4	III
critical self-reflection and lifelong learning	3	IV
5. Health advocacy and social responsibility (Health advocate)		
pharmaco-economics	5	III
ethical and legal responsibility	4	V
6. Leadership and organization (Leader)		
knowledge about quality control and leadership	5	III or IV
organization and quality assurance	9	III or IV
use of information technology	4	V
medication safety	3	V
7. Professionalism (Professional)		
reflection on personal functioning	10	III
integrity, morality, professional behaviour	7	V
Three levels of competency are distinguished, defined as follows. At the end of the master program the student is expected to have the ability to adequately* carry out professional activities … … in a specifically constructed learning situation and/or a simulated professional situation (level III, simulated); … in an authentic professional situation after previous instruction by and under intensive guidance of an experienced pharmacy practitioner (level IV, guided); … in an authentic professional situation (or exceptionally a simulated professional situation) under supervision of an experienced pharmacy practitioner (level V, supervised). Note that levels I (knowledge and skills in standardized situations) and II (knowledge and skills in context-rich situations) are used at intermediate stages only (e.g., bachelor degree).		

* “adequately” means in concord with existing guidelines and/or actual state of (scientific) knowledge.

**Table 2:** Characteristics of the postgraduate specializations.

	Community Pharmacist *	Hospital Pharmacist *
Duration	2 years	4 years
Annual intake	90–140	25–35
Programme content	Centred on task areas of community pharmacists, including patient care, product care and community pharmacy management.	Centred on pharmaceutical patient care (also for patients staying at home to allow shortening of hospital stay), specialized product care (aseptic compounding, radiopharmaceuticals, medical gases) and hospital pharmacy management.
Programme characteristic	Workplace-based learning in one training pharmacy. No individual differentiation. Assessment of competencies based on EPAs.	Workplace-based learning in two training hospitals. Individual differentiation in year 4. Assessment of competencies **
Structure	Programme consists of: workplace based training centralized courses individual and group assignments	Programme consists of: workplace based training centralized courses individual research project individual differentiation in year 4
Centralized courses	Total 24 days.	Total 38 days: 12 (generic, year 1) and 26 (specialized, years 2 to 4).
Workplace	Accredited training pharmacies (n = 350).	Academic (n = 8) and other training hospitals (n = 29).
Guidance and monitoring	One supervisor. Electronic portfolio (custom made).	One trainer and co-trainer per location, several supervisors. Electronic portfolio (EPASS).
Assessment (see Table 3)	CBD/SPO/PE/RW = 14/16/34/16 (80 in total). Format chosen is EPA-dependent.	At least 10/year. SPO, DOPS and CAT used as instruments.
MSF	Once every year.	Three in total: first year and once in each training hospital (years 2 to 4).
FPE	Every three months, structured according to CanMEDS roles.	Every three months, structured according to CanMEDS roles.
Research project	Practice research and development project (group assignment).	Six months (in year 2 to 4). Can be extended to full PhD research project after the specialization.
Intermediate SPE	Suitability (go/no go) assessed after first year. Evaluation by supervisor, decision by director of education.	Suitability (go/no go) assessed after first year. Progress assessed after year 2 and year 3. Evaluation and decision by supervisor.
Final SPE	Decision by director of education, based on advice of supervisor and portfolio review. Specialist status as “community pharmacist” registered by SRC.	Decision by supervisor. Specialist status as “hospital pharmacist” registered by SRC.
Certification of training locations	Certification of location and supervisors by SRC. Obligatory training of supervisors: 2 days	Certification of hospitals and supervisors by NVZA, renewed every 5 years. Obligatory training of supervisors: 2 days.
Governance	Programme developed by KNMP, accredited by the Dutch Board of Pharmacy Specialisms.	Programme developed by NVZA, accredited by Dutch Board of Pharmacy Specialisms.

Abbreviations: KNMP = Royal Dutch Pharmacists Association, NVZA = Dutch Association of Hospital Pharmacists, SRC = Specialist Registration Committee, see Table 3 for other abbreviations. * See refs. [23,40] for details; *** EPAs will be introduced in 2021.

**Table 3:** Authentic feedback and assessment formats.

Assessment Tool	Reference	Description
Objective structured clinical examination (OSCE)	[45,46]	An OSCE usually comprises a circuit of short (usually 5–10 min although sometimes up to 15 min) stations, in which each student/trainee is examined on a one-to-one basis with one or two examiner(s) and either real or simulated patients
Case-based discussion and Entrustment-based discussions (CBD, EBD)	[47,48]	A written report, followed by a short oral discussion with the student/trainee. Used to assess pharmacotherapy related cases; in EBD’s safety risks are especially important.
Critical appraisal of a topic (CAT)	[49,50]	A written report, based on a critical analysis of a case, and supported by a review of the relevant literature.
Short practice observation (SPO) or Directly observed preparation skills (DOPS)	[51]	Observation of work in practice (e.g., a patient consultation, compounding skills, logistic problem solving, teaching activity), which is documented with a judgment
Product evaluation (PE)	[52]	Evaluation by the student/trainee of patient records and other written materials. The quality of the written report is assessed in a structured way
Reflective writing (RW)	[53]	A written self-reflection on a task performed which is afterwards discussed with an assessor or supervisor
Multisource feedback (MSF)	[54,55]	Observations of trainees’ competencies by other pharmacists in the working environment, by pharmacy technicians, general practitioners and patients. Data are collected with an electronic questionnaire, and include a self-assessment by the trainee
Formative performance evaluation (FPE)	[56]	Systematic and structured evaluation of trainee functioning over an extended period, in which all dimensions of performance are taken into account. Focusses on feedback and growth potential. Results are recorded
Summative performance evaluation (SPE)	[56]	As FPE, but aimed at assessing suitability of the trainee for progress in a training program and/or for taking job responsibility (high stakes decisions involved)

**Table 4:** Curriculum elements contributing to professional expertise and identity formation.

Design Principle	Explanation	Master Programs	Specializations
Development of professional expertise (derived from the Integrated Pedagogy Model) *			
1. Support students in their epistemological understanding	Linking scientific knowledge to practical solutions is complex and uncertain. Teachers should help students to deal with these complexities and uncertainties	Students’ practical experiences at the workplace are “debriefed” in reflection meetings during the internships	Interpretation of practical experiences is discussed on a day-to-day basis between trainees and supervisors
2. Provide students with opportunities to differentiate between and among concepts	Students should be repeatedly exposed to relevant concepts in different contexts and should be trained in handling the tools of their profession	Standard operating procedures and protocols are developed and used in courses and internships	Practical problem solving is based on applying procedures and techniques, learned in the undergraduate program
3. Practice with a variety of problems to enable students to experience complexity and ambiguity	Problems should resemble as closely the complexities and ambiguities of their profession. Gradually increase complexity when using problems, cases and representations	Practical application of fundamental concepts is trained in problem-based learning, compounding assignments, and patient-oriented discussions	Complexity and ambiguity are inherently present in day-to-day practical experiences in a community or hospital pharmacy
4. Enable students to understand how particular concepts are connected	Teachers should make explicit connections between facts and concepts and should focus on higher-order (conceptual) learning and “big” concepts	Courses and/or internships are organized around relevant “themes” (e.g., *Polypharmacy* or *Elderly patients*)	Thematic training courses (e.g., *Ethics* or *Pharmacovigilance*) are organized in addition to workplace learning
5. Target for relevance	Teachers should create opportunities for explicit exploration and participation in professional activities	Students have to do individual practice research projects during their internships	Individual assignments (community pharmacy) or a full differentiation project (hospital pharmacy)
6. Share inexpressible knowledge	Expert knowledge can be complex and tacit. Interaction between novices and experts is essential to share this knowledge	Students will observe daily practice and functioning of professionals during their internships	Trainees and experienced colleagues collaborate in daily practice
7. Pay explicit attention to prior knowledge	Educators must be able to recognize, understand and remedy misconceptions of students, stemming from earlier experiences	Teachers in the master programs usually are involved in courses at different levels (also pre-master).	Supervisors and/or trainers can be involved in undergraduate training at universities
8. Support students in strengthening their problem-solving strategies	Teachers should train students in problem solving strategies, relevant for their profession. Explicit guided practice, coaching and/or role modelling is asked for	Specific training (e.g., communication skills) or cognitive skill development (e.g., evidence-based decision making) is part of the curriculum	Specialized practical courses (e.g., Radiopharmaceuticals) are being organized
9. Evoke reflection	Reflection can make tacit knowledge explicit and can improve problem-solving skills and performance. Deep understanding is necessary to avoid “false expertise”	CBD’s require explicit reflection;reflective writing assignments are required as a portfolio constituent	Regular performance evaluations (FPEs) are organized; reflective writing assignments are required as a portfolio constituent
10. Facilitate development of metacognitive knowledge	Students should be aware of their own learning strategies and skills, and limitations. They must learn to monitor, plan and evaluate their own learning	Personal reflection on professional practice are required as a portfolio constituent	Personal development plans are required as a portfolio constituent; MSF requires explicit self-evaluation
Development of professional identity (derived from the Self-determination Theory) *			
A. Need for competence	Fulfilling the need of competence refers to feeling effective and capable of achieving the desired outcomes; frustrating this need refers to feelings of failure	Curriculum design is guided by the CanMEDS framework	Curriculum design is guided by the CanMEDS framework
B. Need for autonomy	Fulfilling the need of autonomy refers to the experience of sovereignty and having choices while carrying out an activity; frustrating this need leads to feeling controlled due to external pressures	Individual research projects, electives and differentiation trajectories accommodate personal development	Individual assignments, elective courses and a formal differentiation (hospital pharmacy) allow personal development
C. Need for relatedness	Fulfilling the need of relatedness refers to the experience of belonging and connection to others, while frustrating this need leads to feeling lonely and excluded	Collaborative, cooperative and project-based educational formats are frequently used; tutorial system is in place	Interaction with peers during centralized courses; frequent interaction with supervisors and other professionals

See * Elvira et al. and Kusurkar et al. for details [14,62].
